# Bridging a genetic gap with new tools to study the metabolism, fitness, and virulence in *Burkholderia cenocepacia*

**DOI:** 10.1128/spectrum.01751-26

**Published:** 2026-09-29

**Authors:** Silvia Buroni

**Affiliations:** 1 Department of Biology and Biotechnology “L. Spallanzani”, University of Pavia 111209, Pavia, Italy; University of Dundee, Dundee, United Kingdom

**Keywords:** *Burkholderia cenocepacia*, genetic tools, metabolism

## Abstract

*Burkholderia cenocepacia* is a formidable opportunistic pathogen, particularly in individuals with cystic fibrosis, yet its study has long been hampered by limited availability of genetic tools due to its high intrinsic antibiotic resistance. A recent study by M. M. Noe, K. Meesilpavikkai, Y. Ajimathorn, A. Sawatpanich, et al. (Microbiol Spectr 14:e02492-25, 2026, https://doi.org/10.1128/spectrum.02492-25) addresses this bottleneck by adapting a unified trimethoprim-selectable toolkit for markerless gene deletion and site-specific chromosomal complementation. By applying these tools to the *purM* gene, the authors demonstrate that while *purM* is universally essential for *de novo* purine biosynthesis, its role in intracellular survival is strain-dependent. These findings highlight the metabolic heterogeneity within the *Burkholderia cepacia* complex and provide a robust methodological framework for future functional genomics to help elucidate the metabolism, fitness, and virulence of these important bacteria. This commentary discusses how these advancements simplify complex genetic workflows and open new avenues for investigating the metabolic determinants of *Burkholderia* pathogenesis and fitness.

## COMMENTARY

The genus *Burkholderia* encompasses a diverse array of species with lifestyles ranging from beneficial plant-associated mutualists to devastating human pathogens. Among these, *Burkholderia cenocepacia* is of particular medical interest, being a critical threat to immunocompromised patients and people with cystic fibrosis because it is capable of causing the so-called “cepacia syndrome”—a life-threatening condition marked by rapid pulmonary decline and sepsis. Despite its clinical importance, the molecular tools available to dissect *B. cenocepacia* features are very difficult to establish. This is mainly due to its large, multi-replicon genome and extensive multidrug resistance profile (1), which often make standard genetic selection markers difficult to use. In this context, the paper by Noe et al. (2) represents a significant advance, taking advantage of *Burkholderia* susceptibility to trimethoprim to enable precise, markerless genetic manipulation and complementation.

## AN EXPANDED TOOLKIT FOR A CHALLENGING PATHOGEN

The primary difficulty in *B. cenocepacia* genetics stems from its notable drug resistance. Most common antibiotics fail to provide the high-stringency selection required for allelic exchange or transposon-based complementation. Although previous successful systems have been developed to study *Burkholderia* genetics (3–7), the system adapted by Noe et al. utilizes a single selection marker—trimethoprim—for both the pEDL1005 vector, set up to create markerless gene deletion, and the mini-Tn7-based complementation system. This approach significantly simplifies cloning procedures and avoids the use of multiple resistance cassettes, which can cause polar effects or limit future genetic studies. Moreover, the use of the mini-Tn7 system (pUC18T-mini-Tn7T-TMP) allows for stable chromosomal complementation downstream of *glmS* homologs and, together with an established PCR-based strategy to verify the site-specific transposon insertion, greatly contributes to improving the genetic toolkit available for this pathogen.

Importantly, the study emphasizes the need for strain-aware optimization, as previously reported (8). For instance, the authors found that *sacB*-mediated counterselection required different sucrose concentrations for the reference strain K56-2 (20%) versus the clinical isolate SCBC075 (25%), although with different yields. This observation must be kept in mind when working with difficult-to-handle microorganisms: even within a single species, physiological and genomic heterogeneity can dictate the success of molecular tools.

## *BURKHOLDERIA* METABOLIC HETEROGENEITY AND PATHOGENESIS

The choice of *purM* (encoding phosphoribosylaminoimidazole synthetase) provides a proof of concept for studying the metabolism of *Burkholderia*, especially in relation to intracellular survival. The authors confirmed that *purM* is essential for *de novo* purine biosynthesis, as Δ*purM* mutants in both tested strains were strict adenine auxotrophs. This aligns with findings in the closely related pathogen *Burkholderia pseudomallei*, where purine limitation in the host environment often renders such mutants avirulent (9).

In contrast, the study reveals once more a fascinating divergence in different *B. cenocepacia* strains regarding intracellular survival. Indeed, while the Δ*purM* mutant of the reference strain K56-2 showed significant defects in early intracellular adaptation within macrophages, the clinical isolate SCBC075 remained fully competent despite its auxotrophy. This suggests that SCBC075 may possess superior purine salvage pathways or more efficient mechanisms for acquiring host-derived nucleotides. This strain-dependent metabolic flexibility underscores that virulence determinants are not monolithic and must be evaluated across diverse genetic backgrounds to understand the pathogenic potential of each strain.

## FUTURE DIRECTIONS

The establishment of the genetic toolkit presented by Noe et al. (2) paves the way for deepening our knowledge of *Burkholderia* strains by providing:

1. **High-throughput functional genomics:** The efficiency of the pEDL1005 and mini-Tn7 systems could be scaled to systematically target metabolic, regulatory, and virulence networks in *Burkholderia* to elucidate fitness and pathogenic pathways not yet completely explored.
2. **Refined complementation strategies:** This study identified a preference for insertion at the *glmS3* locus in K56-2; however, further work is needed to determine if this is the case in other *Burkholderia* species and whether the insertion site influences the expression levels of complemented genes.

In conclusion, the work by Noe et al. provides the *Burkholderia* community with a robust, versatile, and much-needed molecular toolkit. By lowering the technical barriers to genetic manipulation and highlighting the metabolic diversity of *B. cenocepacia*, this research facilitates a new era of functional studies aimed at uncovering therapeutic targets for this recalcitrant pathogen.

## Data Availability

Data will be made publicly available upon publication.

## References

[1] Mahenthiralingam E, Urban TA, Goldberg JB. 2005. The multifarious, multireplicon *Burkholderia cepacia* complex. Nat Rev Microbiol 3:144–156. doi:10.1038/nrmicro1085 15643431

[2] Noe MM, Meesilpavikkai K, Ajimathorn Y, Sawatpanich A, Saninjuk K, Sermswan RW, Chirakul S. 2026. Functional analysis of *purM* in *Burkholderia cenocepacia* using a trimethoprim-selectable allelic exchange and mini-Tn7 complementation approach. Microbiol Spectr 14:e02492-25. doi:10.1128/spectrum.02492-25 PMC13055311 41778813

[3] Flannagan RS, Linn T, Valvano MA. 2008. A system for the construction of targeted unmarked gene deletions in the genus *Burkholderia*. Environ Microbiol 10:1652–1660. doi:10.1111/j.1462-2920.2008.01576.x 18341581

[4] Hamad MA, Skeldon AM, Valvano MA. 2010. Construction of aminoglycoside-sensitive *Burkholderia cenocepacia* strains for use in studies of intracellular bacteria with the gentamicin protection assay. Appl Environ Microbiol 76:3170–3176. doi:10.1128/AEM.03024-09 PMC2869153 20348312

[5] Fazli M, Harrison JJ, Gambino M, Givskov M, Tolker-Nielsen T. 2015. In-frame and unmarked gene deletions in *Burkholderia cenocepacia* via an allelic exchange system compatible with gateway technology. Appl Environ Microbiol 81:3623–3630. doi:10.1128/AEM.03909-14 PMC4421043 25795676

[6] Yap ZL, Rahman ASMZ, Hogan AM, Levin DB, Cardona ST. 2024. A CRISPR-Cas-associated transposon system for genome editing in *Burkholderia cepacia* complex species. Appl Environ Microbiol 90:e00699-24. doi:10.1128/aem.00699-24 PMC11267881 38869300

[7] Scoffone VC, Trespidi G, Barbieri G, Irudal S, Israyilova A, Buroni S. 2021. Methodological tools to study species of the genus *Burkholderia*. Appl Microbiol Biotechnol 105:9019–9034. doi:10.1007/s00253-021-11667-3 PMC8578011 34755214

[8] Logue C-A, Peak IRA, Beacham IR. 2009. Facile construction of unmarked deletion mutants in *Burkholderia pseudomallei* using *sacB* counter-selection in sucrose-resistant and sucrose-sensitive isolates. J Microbiol Methods 76:320–323. doi:10.1016/j.mimet.2008.12.007 19150470

[9] Propst KL, Mima T, Choi KH, Dow SW, Schweizer HP. 2010. A *Burkholderia pseudomallei* Δ*purM* mutant is avirulent in immunocompetent and immunodeficient animals: candidate strain for exclusion from select-agent lists. Infect Immun 78:3136–3143. doi:10.1128/IAI.01313-09 PMC2897367 20404077

